# Restricting the level of the proteins essential for the regulation of the initiation step of replication extends the chronological lifespan and reproductive potential in budding yeast

**DOI:** 10.1007/s10522-024-10113-x

**Published:** 2024-06-06

**Authors:** Karolina Stępień, Tuguldur Enkhbaatar, Monika Kula-Maximenko, Łukasz Jurczyk, Adrianna Skoneczna, Mateusz Mołoń

**Affiliations:** 1 Institute of Medical Sciences, Rzeszów University, 35-959 Rzeszów, Poland; 2 Institute of Biochemistry and Biophysics, Polish Academy of Sciences, 02-106 Warsaw, Poland; 3 The Franciszek Górski Institute of Plant Physiology, Polish Academy of Sciences, 30-239 Krakow, Poland; 4 Institute of Agricultural Sciences, Rzeszów University, 35-601 Rzeszów, Poland; 5 Institute of Biology, Rzeszów University, 35-601 Rzeszów, Poland

**Keywords:** Aging, Cell cycle, Lifespan, Replication

## Abstract

Aging is defined as a progressive decline in physiological integrity, leading to impaired biological function, including fertility, and rising vulnerability to death. Disorders of DNA replication often lead to replication stress and are identified as factors influencing the aging rate. In this study, we aimed to reveal how the cells that lost strict control of the formation of crucial for replication initiation a pre-initiation complex impact the cells’ physiology and aging. As strains with the lower pre-IC control (lowPICC) we used, *Saccharomyces cerevisiae* heterozygous strains having only one functional copy of genes, encoding essential replication proteins such as Cdc6, Dbf4, Sld3, Sld7, Sld2, and Mcm10. The lowPICC strains exhibited a significant reduction in the respective genes’ mRNA levels, causing cell cycle aberrations and doubling time extensions. Additionally, the reduced expression of the lowPICC genes led to an aberrant DNA damage response, affected cellular and mitochondrial DNA content, extended the lifespan of post-mitotic cells, and increased the yeast’s reproductive potential. Importantly, we also demonstrated a strong negative correlation between the content of cellular macromolecules (RNA, proteins, lipids, polysaccharides) and aging. The data presented here will likely contribute to the future development of therapies for treating various human diseases.

**Supplementary Information:**

The online version contains supplementary material available at 10.1007/s10522-024-10113-x.

## Introduction

Replication consists of three main stages: initiation, elongation, and termination. The first stage is to recognize the start site. DNA replication initiates from specific regions on the chromosomes, known as origins of replication (an autonomously replicating sequence (ARS) in *Saccharomyces cerevisiae*) (Rao and Stillman 1995). Replication origins are recognized by the Origin Recognition Complex (ORC) (Li et al. 2018). Not all ARS are used during unperturbed replication; those used are selected by the licensing and activation processes (Remus et al. 2009). One of the factors determining the ARS usage is the formation of the pre-replication complex (pre-RC), i.e., the loading of two MCM complexes onto DNA (Coster and Diffley 2017), an active process that requires ATP hydrolysis and depends on several factors, including the ORC complex and loading factors Cdc6 and Cdt1 (Lewis et al. 2022; Randell et al. 2006). The DNA strand separation needs a DNA helicase activity, which by attaching to ARS and breaking the hydrogen bonds between the bases belonging to two DNA strands, will liberate two strands. However, building the appropriate complex with such a molecular activity requires several additional steps. The activation of the MCM complex’s molecular role occurs in the early S-phase and depends on its assisting proteins and kinases associated with the cell cycle (Bell and Labib 2016; Lewis et al. 2022). Accordingly, Sld3, Sld7 and Cdc45 are recruited to the pre-RC and assembled into the Cdc45-MCM-Sld3 complex with increased levels of Dbf4-dependent kinase (Heller et al. 2011). Next, the S-phase cyclin-dependent kinases phosphorylate Sld3 and Sld2 to promote their binding with Dbp11, which is essential to the formation of the pre-initiation (pre-IC) complex (Muramatsu et al. 2010). The pre-IC complex formation involves the recruitment of several more proteins or complexes (so-called firing factors) and is believed to allow double MCM complex to be separated into single hexamers, as in the active DNA helicase, i.e., is required as switch-on mechanism (Miyazawa-Onami et al. 2017). When the Sld3 is displaced by a GINS complex (Sld5-Psf1-Psf2-Psf3), the CMG (Cdc45-MCM-GINS) helicase complex is formed (Sheu et al. 2016). However, the formation of pre-IC also requires DNA polymerase ε (E) and CDK kinase activity. ADP release and binding of new ATP by MCM leads to CMGE assembly. At the final step, one of the firing factors required for the starting of DNA unwinding, the Mcm10 protein, triggers ATP hydrolysis by CMGE, changing inactive CMG complex into an active DNA helicase resulting in helicase bypass and establishment of replication forks. (Douglas et al. 2018; Lewis et al. 2022).

Thus, through the activity of various essential proteins, among them Sld2, Sld3, Sld7, Dpb11, and Mcm10, the attachment and activation of crucial elements of the replication machinery occur in a strictly depicted order. These factors also determine which initiation sites are fired in a given round of replication (Ilves et al. 2010). As the key component of all replisomes is the major replication DNA helicase, the loading and activating of this helicase by proteins involved in the replication initiation step plays a crucial role in this process (Costa and Diffley 2022).

Disturbances at the different steps of DNA replication and replication stress are often noted as essential factors influencing the aging process. Aging is defined as the gradual deterioration of cellular and organismal functions over time, which increases the risk of improper response to stress and disease susceptibility. A significant overlap exists between the cellular pathways that influence aging and those that contribute to, e.g., neurodegeneration, or metabolic syndrome (de Cabo et al. 2014). DNA replication disorders are also increasingly recognized as a critical factor of genome instability during cancer development (Hills and Diffley 2014; Kotsantis et al. 2018; Macheret and Halazonetis 2015). Obviously, the connections between DNA metabolism and genome maintenance processes, including replication, were also subject of interest for researchers exploring aging mechanisms. However, some processes are difficult to resolve due to the fact that the proteins involved in these processes are essential for life. Approaches that were undertaken mostly rely on mutated alleles of essential genes, which by definition cannot reflect the normal aging process but rather an aging of cells marked by disease. In our study, we asked about the connection between control of the replication initiation process and aging. With time, also during aging, the expression of many genes changes (Frenk and Houseley 2018).

Moreover, it is common knowledge that aging is accompanied by lower proliferating potential. Thus, as a working model, we used the cells with heterozygous loci of essential genes, encoding proteins involved in the initiation of replication (i.e., conditions when expression of these genes is limited). The effect of lowered expression of genes involved in initiating replication on aging is still to be uncovered. Here, we demonstrate that reducing the number of copies of the genes encoding proteins involved in the different steps of initiation of replication, namely *CDC6*, *DBF4*, *SLD3*, *SLD7*, *SLD2*, and *MCM10*, influenced not only respective transcript levels but also the DNA content and integrity of the cell, as well as the proliferative potential and aging of both mitotically active and post-mitotic yeast cells.

## Materials and methods

### Strains and growth conditions

All yeast strains used in this study are in the BY4743 background and are listed in Table 1. The heterozygous strains lacking one functional allele of a given essential gene came from a yeast knock-out collection (Open Biosystems).

**Table 1:** Strains were used in this study

Strain	Genotype	Source
BY4743	*MATa/MATα HIS3/his3Δ1 LEU2/leu2Δ0 LYS2/lys2Δ0 MET15/met15Δ0 URA3/ura3Δ0*	Euroscarf
*CDC6/cdc6Δ*	*MATa/MATα HIS3/his3Δ1 LEU2/leu2Δ0 LYS2/lys2Δ0 MET15/met15Δ0 URA3/ura3Δ0 CDC6/cdc6Δ::kanMX4*	Open Biosystems
*DBF4/dbf4Δ*	*MATa/MATα HIS3/his3Δ1 LEU2/leu2Δ0 LYS2/lys2Δ0 MET15/met15Δ0 URA3/ura3Δ0 DBF4/dbf4Δ::kanMX4*	Open Biosystems
*MCM10/mcm10Δ*	*MATa/MATα HIS3/his3Δ1 LEU2/leu2Δ0 LYS2/lys2Δ0 MET15/met15Δ0 URA3/ura3Δ0 MCM10/mcm10Δ::kanMX4*	Open Biosystems
*SLD2/sld2Δ*	*MATa/MATα HIS3/his3Δ1 LEU2/leu2Δ0 LYS2/lys2Δ0 MET15/met15Δ0 URA3/ura3Δ0 SLD2/sld2Δ::kanMX4*	Open Biosystems
*SLD3/sld3Δ*	*MATa/MATα HIS3/his3Δ1 LEU2/leu2Δ0 LYS2/lys2Δ0 MET15/met15Δ0 URA3/ura3Δ0 SLD3/sld3Δ::kanMX4*	Open Biosystems
*SLD7/sld7Δ*	*MATa/MATα HIS3/his3Δ1 LEU2/leu2Δ0 LYS2/lys2Δ0 MET15/met15Δ0 URA3/ura3Δ0 SLD7/sld7Δ::kanMX4*	Open Biosystems
BY4743 pWJ1344	*MATa/MATα HIS3/his3Δ1 LEU2/leu2Δ0 LYS2/lys2Δ0 MET15/met15Δ0 URA3/ura3Δ0 [RAD52-YFP, LEU2]*	This work
*CDC6/cdc6Δ* pWJ1344	*MATa/MATα HIS3/his3Δ1 LEU2/leu2Δ0 LYS2/lys2Δ0 MET15/met15Δ0 URA3/ura3Δ0 CDC6/cdc6Δ::kanMX4 [RAD52-YFP, LEU2]*	This work
*DBF4/dbf4Δ* pWJ1344	*MATa/MATα HIS3/his3Δ1 LEU2/leu2Δ0 LYS2/lys2Δ0 MET15/met15Δ0 URA3/ura3Δ0 DBF4/dbf4Δ::kanMX4 [RAD52-YFP, LEU2]*	This work
*MCM10/mcm10Δ* pWJ1344	*MATa/MATα HIS3/his3Δ1 LEU2/leu2Δ0 LYS2/lys2Δ0 MET15/met15Δ0 URA3/ura3Δ0 MCM10/mcm10Δ::kanMX4 [RAD52-YFP, LEU2]*	This work
*SLD2/sld2Δ* pWJ1344	*MATa/MATα HIS3/his3Δ1 LEU2/leu2Δ0 LYS2/lys2Δ0 MET15/met15Δ0 URA3/ura3Δ0 SLD2/sld2Δ::kanMX4 [RAD52-YFP, LEU2]*	This work
*SLD3/sld3Δ* pWJ1344	*MATa/MATα HIS3/his3Δ1 LEU2/leu2Δ0 LYS2/lys2Δ0 MET15/met15Δ0 URA3/ura3Δ0 SLD3/sld3Δ::kanMX4 [RAD52-YFP, LEU2]*	This work
*SLD7/sld7Δ* pWJ1344	*MATa/MATα HIS3/his3Δ1 LEU2/leu2Δ0 LYS2/lys2Δ0 MET15/met15Δ0 URA3/ura3Δ0 SLD7/sld7Δ::kanMX4 [RAD52-YFP, LEU2]*	This work
YTE32	*MATa/MATα HIS3/his3Δ1 LEU2/leu2Δ0 LYS2/lys2Δ0 MET15/met15Δ0 URA3/ura3Δ0 RFA1/RFA1-YFP::LEU2*	This work
YTE33	*MATa/MATα HIS3/his3Δ1 LEU2/leu2Δ0 LYS2/lys2Δ0 MET15/met15Δ0 URA3/ura3Δ0 CDC6/cdc6Δ::kanMX4 RFA1/RFA1-YFP::LEU2*	This work
YTE34	*MATa/MATα HIS3/his3Δ1 LEU2/leu2Δ0 LYS2/lys2Δ0 MET15/met15Δ0 URA3/ura3Δ0 DBF4/dbf4Δ::kanMX4 RFA1/RFA1-YFP::LEU2*	This work
YTE35	*MATa/MATα HIS3/his3Δ1 LEU2/leu2Δ0 LYS2/lys2Δ0 MET15/met15Δ0 URA3/ura3Δ0 MCM10/mcm10Δ::kanMX4 RFA1/RFA1-YFP::LEU2*	This work
YTE36	*MATa/MATα HIS3/his3Δ1 LEU2/leu2Δ0 LYS2/lys2Δ0 MET15/met15Δ0 URA3/ura3Δ0 SLD2/sld2Δ::kanMX4 RFA1/RFA1-YFP::LEU2*	This work
YTE37	*MATa/MATα HIS3/his3Δ1 LEU2/leu2Δ0 LYS2/lys2Δ0 MET15/met15Δ0 URA3/ura3Δ0 SLD3/sld3Δ::kanMX4 RFA1/RFA1-YFP::LEU2*	This work
YTE38	*MATa/MATα HIS3/his3Δ1 LEU2/leu2Δ0 LYS2/lys2Δ0 MET15/met15Δ0 URA3/ura3Δ0 SLD7/sld7Δ::kanMX4 RFA1/RFA1-YFP::LEU2*	This work

The *RFA1-YFP* fusion was introduced by yeast transformation with the *RFA1::YFP::LEU2* cassette amplified from the plasmid pRYL24 (Jedrychowska et al. 2019) using primers RFA7317F and RFA6231R (Table 2). The cassette was introduced into one of the *RFA1* loci in the genome of wild-type (WT) (BY4743), *CDC6/cdc6Δ*, *DBF4/dbf4Δ*, *MCM10/mcm10Δ*, *SLD2/sld2Δ*, *SLD3/sld3Δ*, and *SLD7/sld7Δ* strains, respectively. The constructions’ correctness was verified by PCR, using YFP9451R and RFA7367F primers (Table 2).

**Table 2:** Primers used in this study

Primer	Sequence (5ʹ → 3ʹ)
Primers used during strain constructions	
RFA7317F	CAATCGGCTGCTAGCTTAAC
RFA6231R	ACGGTTCACAATCCCTACAG
RFA7367F	GCCGCAACGCAAACTTCATC
YFP9451R	CTTCGGGCATGGCACTCTTG
Primers used for RT-qPCR gene expression analysis	
*ACT1_fw*	AAGCTTTGTTCCATCCTTCT
*ACT1_rev*	GTACCACCGGACATAACG
*DBF4_fw*	AAGCGTCATGAGTAAGAACA
*DBF4_rev*	CTGTGTCTATTTTCCTTTGATGT
*CDC6_fw*	TTTGTCCTGGTTTGAATTGC
*CDC6_rev*	TTTATTTGCAATGTTGGGCC
*SLD2_fw*	GTGAAACGCCAATTAAACTTTC
*SLD2_rev*	GTGGAGGATTAATAGTTGGACT
*SLD3_fw*	CAGACCCTAAAGAGTACATAGAA
*SLD3_rev*	TTTGTAACTGTCACTTCCGT
*SLD7_fw*	ACAACAATCTCAACAAAGGAAG
*SLD7_rev*	GGAGGCCACCCAAAATTAG
*MCM10_fw*	CCGATAATCACAAACGAATTAGA
*MCM10_rev*	TAGGTGGGCGAATTTTAGC

Cells were grown in standard YPD containing 1% Difco Yeast Extract, 2% Yeast Bacto-Peptone, and 2% (*w*/*v*) glucose on a rotary shaker at 150 rpm or on a solid YPD medium containing 2% agar. For strain selection, the SC-Leu medium was used (0.67% Bacto-yeast nitrogen base, 2% (*w*/*v*) glucose, supplemented with adenine and uracil, and all amino acids except leucine). The experiments were carried out at a temperature of 28 °C. Used in chronological lifespan assay, SDC medium contained 0.67% Bacto-yeast nitrogen base (without amino acids) and 2% (*w*/*v*) glucose, supplemented with l-histidine (60 mg/l), l-leucine (180 mg/l) and uracil (60 mg/l).

### Growth rate determination

The growth assays were performed in a liquid medium. Yeast cell suspensions were incubated at 28 °C for 12 h with shaking (Heidolph Incubator 1000 at 1200 rpm). Growth was monitored at 600 nm using the Anthos 2010 type 17,550 microplate reader for 12 at 2 h intervals. In the approach involving counting cells per mL in each culture, a Malassez chamber was used (Carl Roth, Lauda-Konigshofen, Germany).

### Calculation of the mean doubling time

The mean doubling time was calculated for each analyzed strain as described previously (Molon et al. 2016).

### Sporulation efficiency assay

After pre-growing in rich YPD medium, diploid yeast strains were grown for two weeks at 28 °C on sporulation medium (0.1% yeast extract, 1% potassium acetate, 0.05% glucose, 2% agar), as described previously (Krol et al. 2018).

### Flow cytometry analysis

Samples for cytometric analysis were prepared as in (Krol et al. 2018). Briefly, cells were harvested, washed with water, and fixed with a chilled (− 20 °C) 70% ethanol (Polmos, Warsaw, Poland) for 2 h at room temperature. After twice washing with FACS buffer (0.2 M Tris–HCl (Sigma-Aldrich, Burlington, MA, USA), pH 7.4, 20 mM EDTA (Merck, Darmstadt, Germany)), cells were incubated for 2 h at 37 °C in FACS buffer containing 1 mg/ml RNase A (Sigma-Aldrich, Burlington, MA, USA) to digest RNA present in the samples. After washing with phosphate-buffered saline (PBS), cells were stained with 100 μl of propidium iodide solution (50 μg/ml in PBS; Calbiochem, San Diego, CA, USA) overnight at 4 °C in the dark. Just before FACS analysis of the DNA content, 900 μl PBS was added to the cells, and cell suspensions were sonicated three times per 10 s in an ultrasonic bath, Branson 2800 (Branson Ultrasonic Corporation, Danbury, CT, USA) to prevent cells clumping. Analysis was performed using a FACS Calibur (Becton–Dickinson, Franklin Lakes, NJ, USA). A total of 10,000 cells were counted per sample. At least three independent experiments were performed for each strain and selected time points during the Chronological lifespan (CLS) assay. Cells were analyzed with respect to DNA content and cell size using FL2 and FSC channels, respectively. The representative histograms were shown.

### Cell cycle analysis

The cell cycle analysis was performed for exponentially growing cells, as described previously (Stepien et al. 2022), with consideration given to the generation time.

### Determination of budding lifespan

After overnight growth, cells were arrayed on a YPD solid medium plate using a micromanipulator. Budding lifespan was determined microscopically using a micromanipulator, as described previously (Molon and Zebrowski 2017).

### Determination of the total lifespan

The total lifespan was calculated as the sum of reproductive (time between the first and last budding) and post-reproductive lifespans (time between the last budding and cell death), thus showing the length of life of a single mother yeast cell expressed in units of time. The total lifespan of the yeast was determined as previously described by (Minois et al. 2005) with small modifications from (Molon and Zebrowski 2017).

### Chronological lifespan (CLS) assay

The CLS of cells incubated in SDC minimal medium supplemented with necessary amino acids and 2% (*w*/*v*) glucose was measured as previously described (Czachor et al. 2020).

### RNA isolation, reverse transcription and RT-qPCR

The extraction of RNA, reverse transcription, and RT-qPCR were performed as described previously (Stepien et al. 2022). Primers used for quantitative Real-Time PCR are listed in Table 2.

### Determination of Rad52 and Rfa1 foci frequency by fluorescence microscopy

The Rad52-YFP foci formation assay was performed as in (Krol et al. 2018) with some modifications. The studied strains were transformed with the pWJ1344 plasmid carrying a *RAD52::YFP* fusion (Torres-Rosell et al. 2007). The transformants were grown to the exponential phase (about 7 × 10^6^ cells/ml) in a YPD liquid medium at 28 °C with shaking. A 1.5 ml aliquot of each culture was collected, centrifuged (1000 × g), and resuspended in 30 µl of 1 × PBS, and 3.5 µl of cells’ suspension was placed on microscope slides to assess the percentage of cells spontaneously forming Rad52-YFP foci. To the remaining culture, zeocin was added to the final concentration of 100 μg/ml, and the cells were incubated for an additional hour under the same conditions. Then, an aliquot of zeocin-treated strains was collected to examine the percentage of cells forming stress-induced Rad52-YFP foci. Imaging was performed at 100-fold magnification in DIC and YFP channels of an Olympus BX-51 fluorescent microscope operated by cellSens Dimension software and documented using a DP-72 camera. The numbers of cells and Rad52 foci in the cells were counted, and the average percentage of cells with Rad52 foci was calculated after screening at least 300 cells in each of three biological repeats for a total count of at least 900 cells. The results are presented as the quartiles of data, with the mean, median, SD, and *p*-values calculated using a two-sample Welch *t*-test.

The Rfa1-YFP foci were analyzed using a similar experimental scheme as described above, except that the strains with *RFA1-YFP::LEU2* fusion in the genomic locus of *RFA1* were used. Imaging was performed using a Zeiss AxioCam MRc5 Digital Camera (Zeiss, Oberkochen, Germany), mounted on a Zeiss Axio Imager.M2 fluorescence microscope operated by Zeiss Axio Vision 4.8 software, using DIC (for bright field) and 38HE filter set (for YFP).

Since, in opposite to Rad52-YFP foci, the Rfa1-YFP foci rarely occur single per cell, we adopted semi-automatic counting of their number according to the methodology applied for another repair foci, Rad51-YFP, and described in (Antoniuk-Majchrzak et al. 2023). In brief, the Rfa1-YFP foci number was counted using several image-processing software, as follows: (1) the binary masks of cells were obtained through Cellpose software (RRID:SCR_021716, (Stringer et al. 2021); (2) the binary masks of Rfa1 foci were produced by image preprocessing in Fiji ((RRID:SCR_002285, (Schindelin et al. 2012)) with the plugin MorphoLibJ (Legland et al. 2016); (3) the resulted images were used to generate probability masks using semantic segmentation in ilastik (Berg et al. 2019) (4) then, the occurrences of respective foci in the areas of individual cells were counted using CellProfiler software (Stirling et al. 2021). At least 600 cells were analyzed in each of the three biological repeats. The results are presented as the quartiles of data, with the mean, median and SD marked. The statistical significance of the results was tested by a two-sample Welch *t*-test.

### *DAPI staining of mitochondrial DNA in the *in vivo* assay*

Cells grown in YPD medium to the exponential phase at 28 °C with shaking were per additional 1 h cultivated in the same conditions but in the dark with the addition of 4ʹ,6- diamidino-2-phenylindole (DAPI; Invitrogen) to a final concentration 1 μg/ml. Then, cells were pelleted by centrifugation (800 g), washed twice in PBS, suspended in 50 μl PBS and placed on a microscope slide. Imaging was performed using a Zeiss AxioCam 807c Digital Camera (Zeiss, Oberkochen, Germany), mounted on a Zeiss Axio Imager.M2 fluorescence microscope operated by Zeiss ZEN software, using DIC (for bright field) and 49 filter set (for DAPI).

Analysis of mtDNA fluorescent signals was performed similarly to the Rfa1 foci analysis described above. After image deconvolution, the binary masks for cells and mtDNA signals were prepared and used for segmentation. Fluorescent signals’ intensities were calculated by converting mtDNA binary masks to regions of interest in Fiji software and calculating the mean integrated density value for each set of raw images. At least 900 cells were analyzed in each of the three biological repeats. Data for all mtDNA were counted for each strain. Statistical significance was calculated using the Welch t-test.

### Raman spectroscopy

Lyophilized yeast samples of WT and lowPICC strains were used to analyze their chemical composition using the FT-Raman Nicolet NXR 9650 spectrometer equipped with a 1064 nm laser. FT-Raman spectra were measured at an aperture of 50 and a spectral resolution of 8 cm^−1^. The spectra were recorded in the range of 300–3.500 cm^−1^ with a laser power of 0.5 W, and the diameter of the laser beam was 50 μm. For each spectrum, 64 scans were collected. Measurements were made in eight replicates. Raman spectra were processed by the Omnic and OriginLab software.

The principal components analysis (PCA) was used to compare samples for similarities and differences in Raman ranges for lipids, polysaccharides, proteins, and RNA.

### Statistical analysis

The results represent the mean ± SD values for all tested samples in two independent experiments. The differences between the WT and isogenic heterozygous diploid strains were estimated using one-way ANOVA and Dunnett’s post hoc tests. The values were considered significant when *p* < 0.05. The statistical analysis was performed using the Statistica 10.0 software, and statistical and multidimensional analysis was conducted using PAST 3.0, Origin 2018 software (Raman spectroscopy), and OriginPro (fluorescence microscopy).

## Results and discussion

### Reducing the number of genes involved in the regulation of initiation of DNA replication causes disturbances in DNA content and growth rate

The way to resolve the role of essential genes in various biological processes without changing their molecular function, which excludes the usage of the point mutants, is to use the conditions in which their expression would be limited. One of the ways to obtain such an experimental model is the usage of heterozygous strains that possess only one copy of the essential gene. For some time, we have been studying the far-reaching connections between proteins involved in the initiation of replication and the cell aging process, which also leave marks on cellular metabolism (Stepien et al. 2022, 2024). In the present project, we follow up on the influence on cells’ lifespan and the factors involved in the initiation step of replication. This time, however, we are not interested in factors that recognize ARS sequence as the ORC complex does or that provide the ability to unwind the DNA helix as the CMG helicase complex does. We are interested in how the other factors involved in the initiation of the replication step, e.g., these modulating the molecular function of ORC and CMG, enabling the formation of functional pre-IC, thus, factors responsible for the control of the initiation of replication, influence the cells’ lifespan. Thus, in the present study, we concentrated on the following genes: *CDC6*, *DBF4*, *SLD3*, *SLD7*, *SLD2*, and *MCM10*, and to be able to study phenotypes, we used the yeast strains heterozygous with respect to these genes. For ease of reference, we will refer to lower pre-IC control (lowPICC) strains when writing about strains: *CDC6/cdc6Δ*, *DBF4/dbf4Δ*, *SLD3/sld3Δ*, *SLD7/sld7Δ*, *SLD2/sld2Δ*, and *MCM10/mcm10Δ*.

**Fig. 1:**
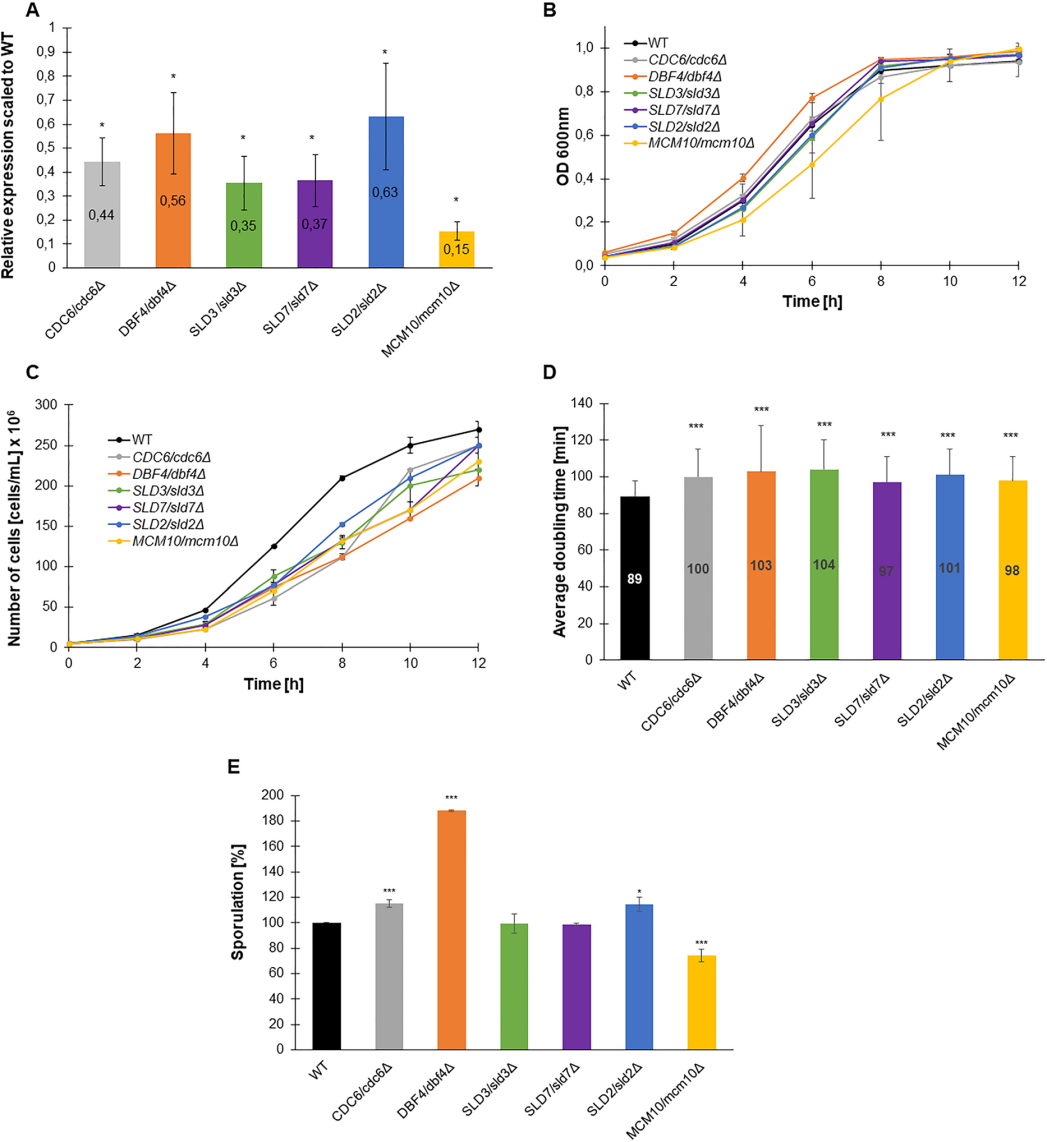
Phenotypic characterization of lowPICC strains. Growth and sporulation efficiency phenotypes of strains lacking one copy of the gene encoding proteins are necessary for the control of the initiation of replication. The relative expression ratio of *CDC6*, *DBF4*, *SLD3*, *SLD7*, *SLD2*, and *MCM10* normalized to *ACT1* and to the expression level in WT in the respective heterozygous strains was calculated from five independent biological repetitions (**A**). Comparison of growth curves for respective heterozygous lowPICC strains and WT control (BY4743) determined by the optical density (**B**) or number of cells per ml (**C**). An average doubling time of single yeast mother cells is estimated during the budding lifespan. The error bars indicate standard deviations from two independent experiments (**D**). Sporulation frequency of the BY4743 and lowPICC strains (**E**). Standard deviation was also counted. Data are expressed as mean ± SD from three independent experiments. Bars indicate SD. Statistical significance was assessed using ANOVA and Dunnett’s post hoc test (*p < 0.05; **p < 0.01; ***p < 0.001) compared to the WT

We started by ensuring that, indeed, in the lowPICC strains, the expression level of respective genes was lowered. Using the quantitative-RT-PCR approach, we determined the expression levels of respective genes in all lowPICC heterozygous strains as compared to their expression in the WT (BY4743) strain. Results shown in Fig. 1A proved the significant reduction (*p* < 0.05) in the expression of all tested genes in the respective lowPICC strain compared with the expression level of the same gene in a WT strain. The most pronounced decrease in expression was observed for the *MCM10* gene in the *MCM10/mcm10Δ* strain. Thus, all heterozygous, lowPICC strains could serve in further experiments to follow the phenotypic effects of lower expression of assayed genes.

We compared the growth rate and the average doubling time of the set of analyzed heterozygous lowPICC strains on a rich medium with 2% glucose. Two methods were used to estimate the growth rate: changes in optical density and the number of cells per mL in cultures during the experimental period. Both measurements results were shown because, in comparison to the growth rate analysis (Fig. 1B, C), the doubling time analysis revealed a statistically significant extension of the cell cycle (Fig. 1D). It has been suggested that this phenotype might be associated with changes in cell size, morphology, transparency or thickness of the cell wall of analyzed strains. As was shown in Fig. 1C, all tested strains presented a flatter steep growth curve than the WT control, which suggested expanded doubling time. The most pronounced effect was seen for *DBF4/dbf4Δ* and *SLD2/sld2Δ*. As shown in Fig. 1D, the mean cell doubling time increased significantly for all lowPICC strains compared to WT (*p* < 0.001). The average doubling time of a single cell was calculated during a routine reproductive potential analysis, which is the most accurate method of estimating doubling time in budding yeast. It disregards variations in cell size and minimizes the effect of virgin cells (constituting 50% of the population in the exponential phase).

In our previous studies, we documented that the lowered expression of genes encoding the ORC or CMG complexes’ subunits results in a significant decrease in the growth rate (Stepien et al. 2022, 2024). Thus, the current results followed the previously shown rule that the exact controlled expression level of essential genes encoding proteins involved in the initiation of replication is necessary to maintain the WT growth rate phenotype.

We also examined sporulation efficiency in the lowPICC strains. As shown in Fig. 1E, most of the tested strains exhibited altered sporulation efficiency, except *SLD7/sld7Δ* and *SLD3/sld3Δ*, which behave exactly as WT. However, not all strains displayed the sporulation efficiency change in the same direction, e.g., in the *DBF4/dbf4Δ* strain, it increased by about 80%; in *SLD2/sld2Δ* and *CDC6/cdc4Δ* strains, mild growth in sporulation efficiency was noticed, while in the *MCM10/mcm10Δ* strain decrease by about 20% in the sporulation efficiency was observed. Those data suggest that aberrations in proper control of the initiation replication step also impact meiosis. Since the lowered level of expression of genes encoding factors influencing replication initiation does affect the effectiveness of the sporulation process, we can assume the mutations in the respective genes could impact yeast’s ability to sporulate as well, if only by leading to changes in the speed of this process. Consequently, this observation may serve as a starting point for further research to understand these particular connections and reveal mechanisms controlling this process.

**Fig. 2:**
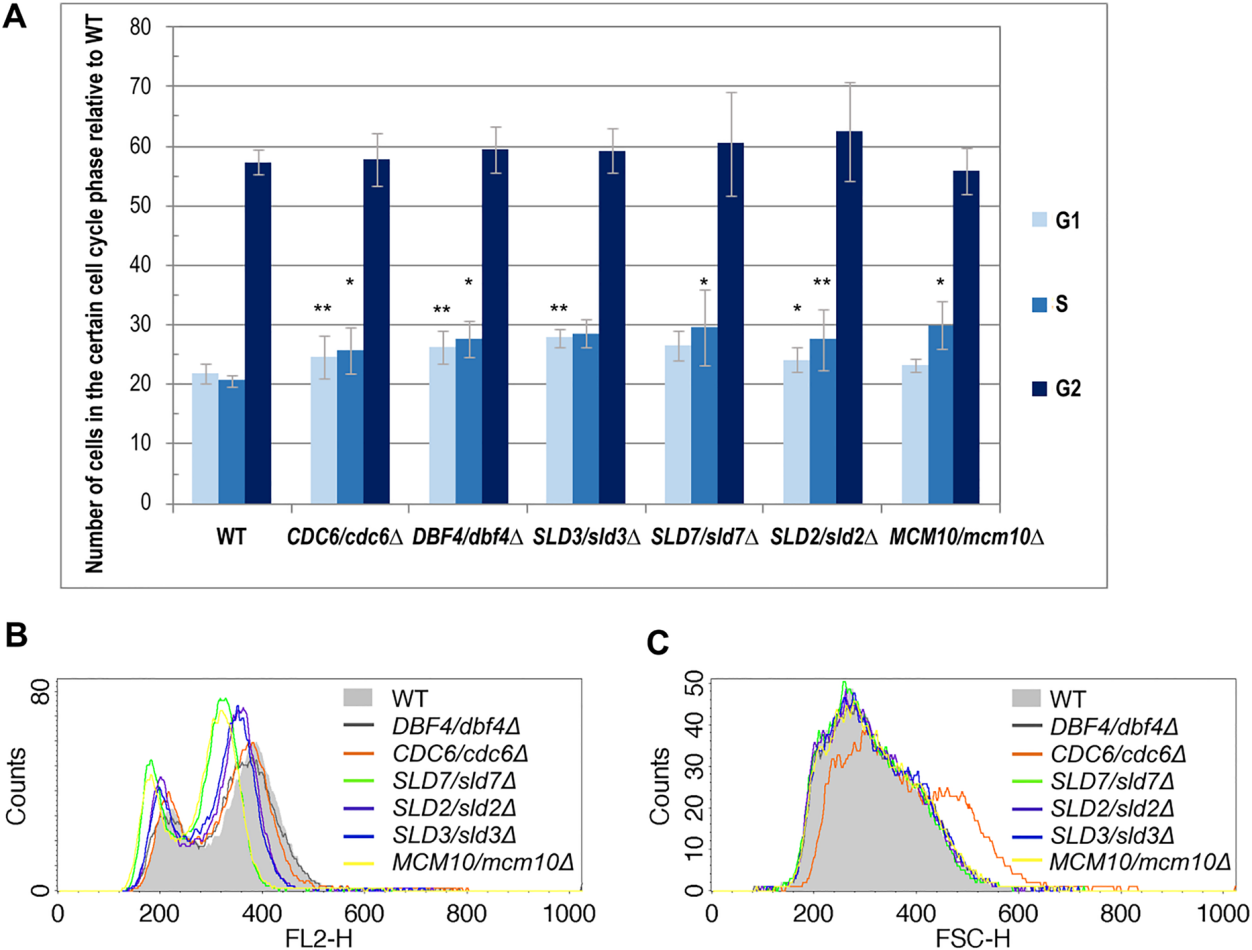
Flow cytometry analysis revealed cell cycle aberrations in lowPICC strains. The cells of WT (BY4743) and the isogenic heterozygous strains *DBF4/dbf4Δ*, *CDC6/cdc6Δ*, *SLD7/sld7Δ*, *SLD2/sld2Δ*, *SLD3/sld3Δ*, and *MCM10/mcm10Δ* strains, grown at 28 °C in YPD medium to the exponential phase, were labeled with propidium iodide and their DNA content was tested using flow cytometry. **A** The quantification of the cell cycle analysis results. The percentage of cells in the specific cell cycle phase was calculated considering the strains’ generation time and shown with respect to WT. The mean of three biological replicates is shown. Bars indicate standard deviations. Statistical significance with respect to the cell cycle phase of the WT control was assessed using the Student’s t-test (*p < 0.05; **p < 0.01). For the gating conditions, see supplementary Fig. S1. The DNA content and (**B**) and cells’ size (**C**) histograms (mean of three biological repetitions) of lowPICC strains versus WT were shown

The initiation of replication must be tightly controlled in order to ensure that the entire genome is duplicated precisely in each cell cycle. This is realized by coordinating the first steps in DNA replication, contributing to the replication initiation: ARS recognition, the successive building of pre-IC, and activating the replicative DNA helicase. Therefore, we investigated if and how the cell cycle changes in lowPICC strains’ cells. We used flow cytometry analysis of propidium iodide-stained cells to reveal the potential cell cycle abnormalities. As shown in Fig. 2A (Fig. S1), a slight increase in the length of the G1 and S-phase of the cell cycle were detected in most of the assayed strains. Both cell cycle phases were prolonged in *CDC6/cdc6Δ*, *DBF4/dbf4Δ*, and *SLD2/sld2Δ* strains. In the *SLD3/sld3Δ* strain cells, the G1 phase was elongated significantly (by 27%), while the S phase increased the most of all tested strains in the *SLD7/sld7Δ* and *MCM10/mcm10Δ* strains, by 43% and 45%, respectively.

Interestingly, flow cytometry analysis also showed differences in the DNA content among tested strains (Fig. 2B). A significant increase in the size of the cells in the *CDC6/cdc6Δ* strain population was also noticed (Fig. 2C). These data suggest that the differences in the growth rate of the analyzed strains do not result simply from the cell cycle length changes, but other factors might play a role there. Considering the effect of decreased expression of genes encoding proteins engaged in the initiation of DNA replication on the cell cycle phases’ length, we saw the following rule. The most significant impact on the G1 phase duration, i.e., its significant elongation, was observed when the ORC subunits gene expression level declined (Stepien et al. 2022); a lower effect was seen in the strains with a decreased expression of the CMG helicase subunits (Stepien et al. 2024), and the yet minor but more diverse (as concerning both, G1 and S phases of the cell cycle) effect, was shown in this work, for lowPICC strains.

In eukaryotes, chromosome replication starts from multiple origins, which are activated at different time points during the S phase and terminates when converging replication forks meet (Hyrien and Goldar 2010). Even though we already know there are early and late types of origins and that the origin chromosomal localization, active transcription, or occurrence of DNA damage might influence the origin usage during replication, the rules that guide the origin licensing and firing are far from being solved (Early et al. 2004; Eshaghi et al. 2007; Legouras et al. 2006; Zappulla et al. 2002). At present, we believe that differences in doubling times in analyzed heterozygous strains with limited availability of proteins involved in the control or activation of replication initiation may result from a changed pattern of replication start sites used. Previous research has proven that some factors involved in the initiation of DNA replication in yeast are also responsible for regulating both the origin sites chosen and the time activated in a replication round. Therefore, yeast origin firing is an important part of cell cycle regulation, and as was demonstrated previously, the proper programmed of origin firing prevents incorrect checkpoint activation and regulates the length of the S-phase in budding yeast (Mantiero et al. 2011). The activation efficiency of the origin sites increased genome-wide when Sld2 and Dbf4 proteins were overexpressed simultaneously with Cdc45 and Sld7 proteins (McGuffee et al. 2013). Additionally, Tanaka et al. found that overexpression of only Sld3, Sld7, and Cdc45 could speed up the activation time of origins that would usually be fired as last (Tanaka et al. 2011a). The disturbed growth rates may also be related to disorders at the level of MCM helicase loading caused by a decrease in the level of, e.g., Cdc6 or ORC, which makes licensing difficult (Kotsantis et al. 2018).

The finding that some specific replication initiation factors, e.g., Sld2, Sld3, and Sld7, are expressed at levels significantly lower than the pre-RC and replisome components suggests that they are crucial for origin activation and successful initiation of replication (Mantiero et al. 2011). In the case of the analyzed set of lowPICC strains, changes in doubling time are also associated with slight cell cycle disorders; however, we should stress here that two cell cycle phases were affected in most of them, the G1 and S phases. These results are supported by the observation of similar effects for various deletions or conditional mutants in these genes. The prolongation of G1 and/or S phases was noticed, e.g., in *mcm10ΔC* (Douglas and Diffley 2016), *sld7Δ* (Tanaka et al. 2011b), *sld3-*5 (Kamimura et al. 2001), *sld2-5td* and *sld3-2A* (Tanaka et al. 2007), *cdc6*^K114A^ (Weinreich et al. 1999), and in strain carrying *TetO*_*7*_*-DBF4* in the shut off conditions for *TetO* promoter (Yu et al. 2006).

Additionally, flow cytometry analysis showed the DNA content differences between some of lowPICC strains and WT control (Fig. 2B). The two groups with lower than WT control DNA content were visible; the *SLD2/sld2Δ* and *SLD3/sld3Δ* strains showed slight shifts, and the *SLD7/sld7Δ* and *MCM10/mcm10Δ* strains showed more significant shift on the FL2-H axis to the left, suggesting drop down in the fluorescence intensity of DNA intercalating dye (here, propidium iodide). Such a shift is usually interpreted as a decrease in the DNA content, e.g., due to increased DNA damage, the error-prone DNA repair leading to DNA rearrangement events resulting in the loss of part or even whole chromosomes. However, it can also be attributed to the higher DNA condensation or accumulation of single-stranded DNA regions that limit the intercalation of fluorescent dye into DNA. Another source of DNA content shift might be a lowered number of mitochondrial DNA (mtDNA). The result was so striking that we decided to have a closer look at this issue.

### Lowered expression of lowPICC genes leads to aberrant DNA damage response and affects mtDNA content

**Fig. 3:**
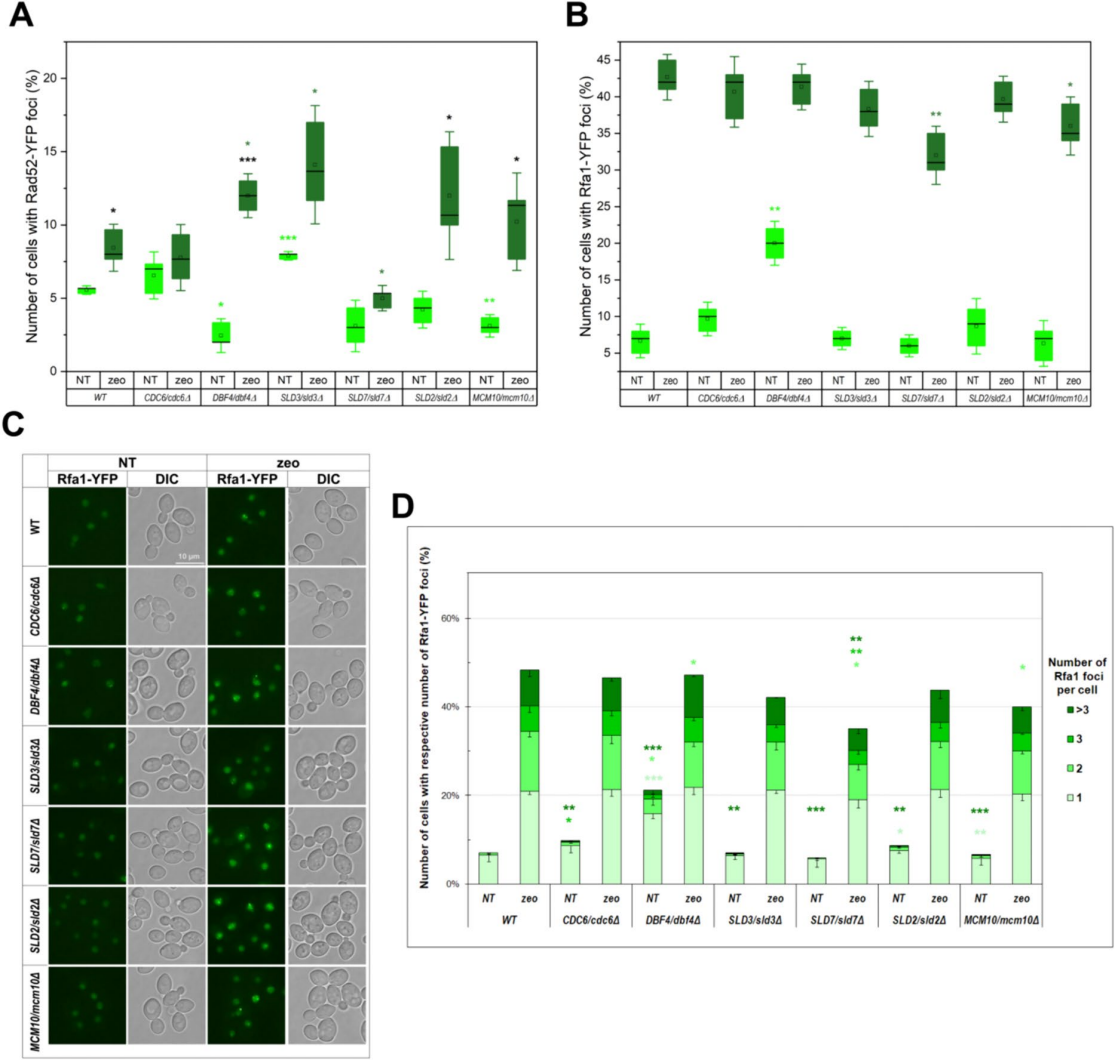
Analysis of the frequency of DNA double-strand breaks and ssDNA region in lowPICC strains. The Rad52-YFP and Rfa1-YFP foci were detected in WT and the isogenic heterozygous strains *DBF4/dbf4Δ*, *CDC6/cdc6Δ*, *SLD7/sld7Δ*, *SLD2/sld2Δ*, *SLD3/sld3Δ*, *MCM10/mcm10Δ* grown to exponential phase (see material and methods section for details). The Rad52-YFP (**A**) and Rfa1-YFP (**B**) foci frequency quantification. Three biological replicates were performed, each with at least 300 cells (for Rad52) or 600 cells (for Rfa1) counted for every strain and condition. Boxes represent the quartiles of data. Horizontal lines in the boxes represent the median values. The square represents the mean value. Whiskers represent standard deviation with a coefficient = 1.5. Statistical hypothesis testing was conducted using a two-sample Welch *t*-test. *p < 0.05; **p < 0.01, ***p < 0.001. The light green stars reflect the statistical significance of observed difference with respect to the non-treated control strain, the dark green stars reflect the statistical significance with respect to the zeocin-treated control strain, and the black stars reflect the statistical significance of change observed for a single strain between treatment with zeocin and control conditions. For clarity, the statistically significant difference (*p* < 0.001) in the Rfa1 foci number between non-treated and zeocin-treated cells was omitted from the graph (**B**). **C** Rfa1-YFP foci were detected in the same strains as in (**A** and **B**). **D** The graph shows the quantification of the number of the cells with respective Rfa1-YFP foci number per cell. The mean (± SD) of the percentage of cells with one, two, three or more Rfa1-YFP foci in cells were counted in three independent biological repetitions. The statistical significance of the difference between the WT control strain and a given lowPICC strain was shown above the result for the given strain in control conditions. Alike was done for the results obtained for strains treated with zeocin. The difference in the percentage of cells with the particular number of Rfa1-YFP foci per cell between non-treated and zeocin-treated strains was statistically significant (*p* < 0.01) for each strain and thus was omitted from the graph. The stars representing the statistical significance of the results for the certain subpopulation of the cells are shown in the same color as this population

To reveal if lowPICC strains accumulate DNA damage or ssDNA regions, we combined fluorescence microscopy with the usage of fluorescently labeled proteins, Rad52-YFP (a recombinase involved in DNA damage repair via homologous recombination) and Rfa1-YFP (a subunit of the ssDNA binding RPA complex), that are widely used markers in such applications (Lisby et al. 2001; Ngo et al. 2020). The results of these experiments are shown in Fig. 3.

The number of cells with spontaneously formed Rad52-YFP foci increased significantly in the strain *SLD3/sld3Δ*, while it decreased significantly in *DBF4/dbf4Δ* and *MCM10/mcm10Δ* strains (Fig. 3A). Zeocin treatment, which results in double-strand stress, usually causes an increase in the percentage of cells with Rad52-YFP foci because the recombinase Rad52 is recruited to the damage site, where the DNA repair occurs. Indeed, that is what was observed for the WT control, as well as for most of the strains, but with some exceptions. The *CDC6/cdc6Δ* strain almost did not react to zeocin-stress. In the *SLD7/sld7Δ* strain, the increase in the number of cells with Rad52-YFP foci formed after zeocin treatment was neglectable, but what caught our attention was that the number of cells with the stress-induced foci in this strain was significantly lowered compared to their number in the control strain treated with zeocin. Altogether, the observed phenotypes were highly variable. In two strains, *DBF7/dbf4Δ* and *SLD3/sld3Δ*, the number of stress-induced Rad52-YFP foci rose significantly compared to zeocin-treated control. At the same time, the percentage of cells spontaneously forming Rad52 foci in those strains differed almost fourfold. In the other two strains, *SLD2/sld2Δ* and *MCM10/mcm10Δ*, the percentage of stress-induced Rad52-YFP foci rose significantly compared to non-treated control for respective strains, but the raising range varied (about two and four-folds, respectively).

As shown in Fig. 3B–D, in the control conditions, the number of cells with Rfa1-YFP foci was significantly increased in the *DBF4/dbf4Δ* strain compared to the WT. Moreover, in the population of cells containing Rfa1-YFP foci, more cells containing several foci per cell were present (in contrast to Rad52-YFP foci, which mostly appear single and only rarely as two per cell). Such a phenotype frequently accompanies increased DNA damage (see Fig. 3D) or accumulation of ssDNA gaps. Interestingly, even if the frequency of cells with Rfa1-YFP foci did not change, all the rest of the lowPICC strains displayed an increased number of cells with a high number (more than three) Rfa1-YFP foci per cell, which was not observed in the control strain. The zeocin treatment, which causes the double-strand break induction, resulted in a significant increase in the Rfa1-YFP-containing cell population, including an increase in the foci number per cell. Furthermore, the number of cells containing zeocin-induced Rfa1-YFP foci decreased significantly in the *SLD7/sld7Δ* and *MCM10/mcm10Δ* strains, mainly those with multiple foci.

Described phenotypes helped us understand possible sources of DNA content shifts observed for some of the tested strains. For example, the accumulation of Rad52-YFP foci-containing cells in the *SLD3/sld3Δ* strain suggested more frequent DNA damage in those cells and subsequent higher requirements for homologous recombination. Since the Rfa1-YFP foci did not accumulate in this strain, we can assume the double-strand break repair pathway is activated in those cells. In the strains with increased double strand breaks (DSBs), the risk of loss of part of the repetitive sequence rises. One of the naturally occurring repetitive sequences in the genome is the region encoding rRNA, which in yeast is located on chromosome XII arm. Since repetitive sequences and highly expressed DNA are risk factors promoting genome instability, and rDNA unquestionably belongs to both groups, the rDNA region of chromosome XII is tough to maintain; thus, its aberration serves frequently as a marker for genome instability. The average number of rDNA repeats on this chromosome arm is around 125 in the WT strain. The length of individual rDNA units in *S. cerevisiae* is 9.1 kb, so an average rDNA array is more than 1.1 Mbp long. In the case of long repetitive sequences such as rDNA repeats, the loss of their part could be detectable even in DNA content analysis. The strains vary in the number of repetitions, and the lower rDNA array length results in generation time elongation.

We showed the *SLD3/sld3Δ* strain has prolonged generation time, displayed a high frequency of recombinase Rad52 recruitment to the DNA damage sites, and that the DNA content of this strain decreased. These results are in line with the data shown in (Lynch et al. 2019), showing the association of Sld3 depletion with chromosome XII instability. The authors showed a similar correlation with Sld2 depletion, but while we do see the increase in generation time and a higher percentage of cells with Rad52-YFP foci after zeocin treatment, in contrast to *SLD3/sld3Δ*, the spontaneous forming Rad52-YFP foci number did not increase in the *SLD2/sld2Δ* strain. Therefore, the connection is not so obvious in this case and would require further investigation to reveal the mechanism of rDNA array instability in that strain. However, it should be mentioned that a previous study has reported that yeast strains with reduction-of-function alleles of *SLD2* and *MCM10*, *SLD3*, *DBF4*, and *CDC6* displayed chromosome instability phenotypes (CIN) (Stirling et al. 2011). The increased frequency of gross chromosomal rearrangements (GRC) was shown for the first four strains, and increased chromosome transmission fidelity was shown for *cdc6-1*. Moreover, a strain with depleted *SLD2* level and *mcm10-1* mutant were qualified as strains with strong CIN phenotype.

The *mcm10-1* strain is repetitively shown on the screens for genomic unstable mutants. For example (Su et al. 2015) showed in the *mcm10-1* mutant cells CAG tracts instability, which, as the authors believe, relies on the increased Slx5/8-dependent SUMOylation of Rad52 bound to the CAG tract, which targets this recombinase to degradation. Moreover, (Thu et al. 2016) showed, in the *mcm10-1* strain, increased sumoylation of several Slx5-Slx8 SUMO-targeted ubiquitin ligase (STUbL) substrates, among them Rad52, Rad59, Sgs1, i.e., proteins important for homologous recombination repair. As was shown lastly for another protein crucial for homologous recombination, recombinase Rad51, posttranslational modification with SUMO is crucial for Rad51 recruitment to DNA, while its ubiquitination by STUbL E3, Slx5-Slx8 complex is indispensable for repair foci dissolution, which allows to finish repair (Antoniuk-Majchrzak et al. 2023). Thus, due to an imbalance in the SUMOylation level of homologous recombination involved protein in the *mcm10-1* strain, the faithful DNA damage repair could be staggered, leading to favor of the error-prone pathways of HR (e.g., SSA or BIR). In effect, in the *mcm10-1* strain, likewise in the strains lacking Slx5 or Slx8, the gross chromosomal rearrangements (in the case of *slx5Δ*, predominantly deletions) are elevated, and telomere length is affected (Nagai et al. 2008; Zhang et al. 2006).

**Fig. 4:**
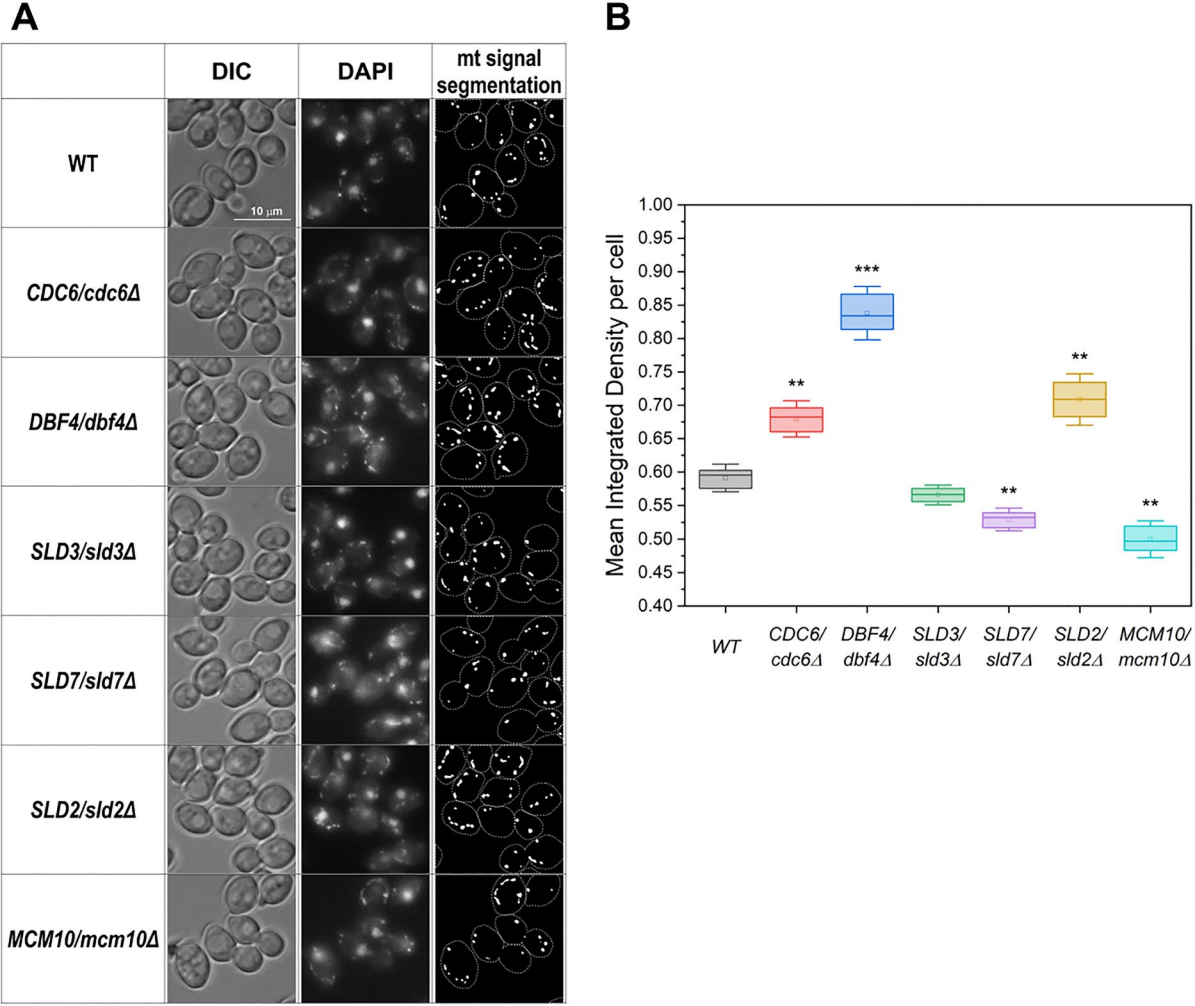
Changes in the mtDNA content in the lowPICC strains’ cells. The WT control and heterozygous strains *DBF4/dbf4Δ*, *CDC6/cdc6Δ*, *SLD7/sld7Δ*, *SLD2/sld2Δ*, *SLD3/sld3Δ*, *MCM10/mcm10Δ* grown to exponential phase were stained with DAPI in vivo. The DAPI signal was documented by fluorescent microscopy. Then, the segmentation of cells and mtDNA signals in the pictures obtained was performed, and the integrated density of mtDNA signals per cell was measured. **A** Illustration of DAPI staining results of lowPICC strains and an example of segmentation of both cells (marked with a line) and mtDNA signal (bright spots). **B** Graph showing quantified results of mtDNA fluorescent signal intensity displayed as the mean integrated density of segmented mtDNA signal per cell (i.e., the sum of the values of the pixels in the area of segmented mtDNA). At least 300 cells per each from three independent biological repetitions were analyzed. Boxes represent the quartiles of data. Horizontal lines in the boxes represent the median values. The dot represents the mean value. Whiskers represent standard deviation with a coefficient = 1.5. The statistical significance of the results was checked using the Welch t-test. *p < 0.05, **p < 0.01, ***p < 0.001

Since mtDNA level might also affect total cellular DNA content, we performed an experiment that allowed the measurement of mtDNA content. We stained the lowPICC strains’ cells with DAPI and analyzed the mtDNA-derived fluorescent signal. Figure 4 summarizes the results of this experiment. Only the *SLD3/sld3Δ* strain displayed mtDNA content close to that observed in the control strain. The *DBF4/dbf4Δ*, *SLD2/sld2Δ*, and *CDC6/cdc6Δ* strains showed mtDNA accumulation, whereas in *DBF4/dbf4Δ* strain the mtDNA content increased by 40%, and in the other two strains by 22 and 13.5% respectively (Fig. 4A, B). In the *MCM10/mcm10Δ* and *SLD7/sld7Δ* strains, we observed the opposite effect; the mtDNA content decreased by 17% and 11%, respectively. This result helps explain previously obtained results, such as shifts in DNA content histograms. The strains with the lowest DNA content are actually the same as those with the lowest mtDNA content. The higher mtDNA content masks to some extent the cellular DNA content aberrations resulting from, e.g., high frequency of ssDNA regions in *DBF4/dbf4Δ* strain cells or high number of DNA damage that may lead to DNA rearrangements or DNA loss in *SLD2/sld2Δ* strain cells. The changes in the mtDNA detected in the lowPICC strains are significant because the previously published data indicated the connection between mtDNA level and various biological processes contributing to genome stability (Puddu et al. 2019). For example, the mtDNA level increases in the strains that have activated the DNA damage response and are accumulating dNTPs. The loss of mtDNA was correlated to aneuploidy.

### Lowered expression of respective essential genes in lowPICC strains affects cells’ reproductive potential and aging

**Fig. 5:**
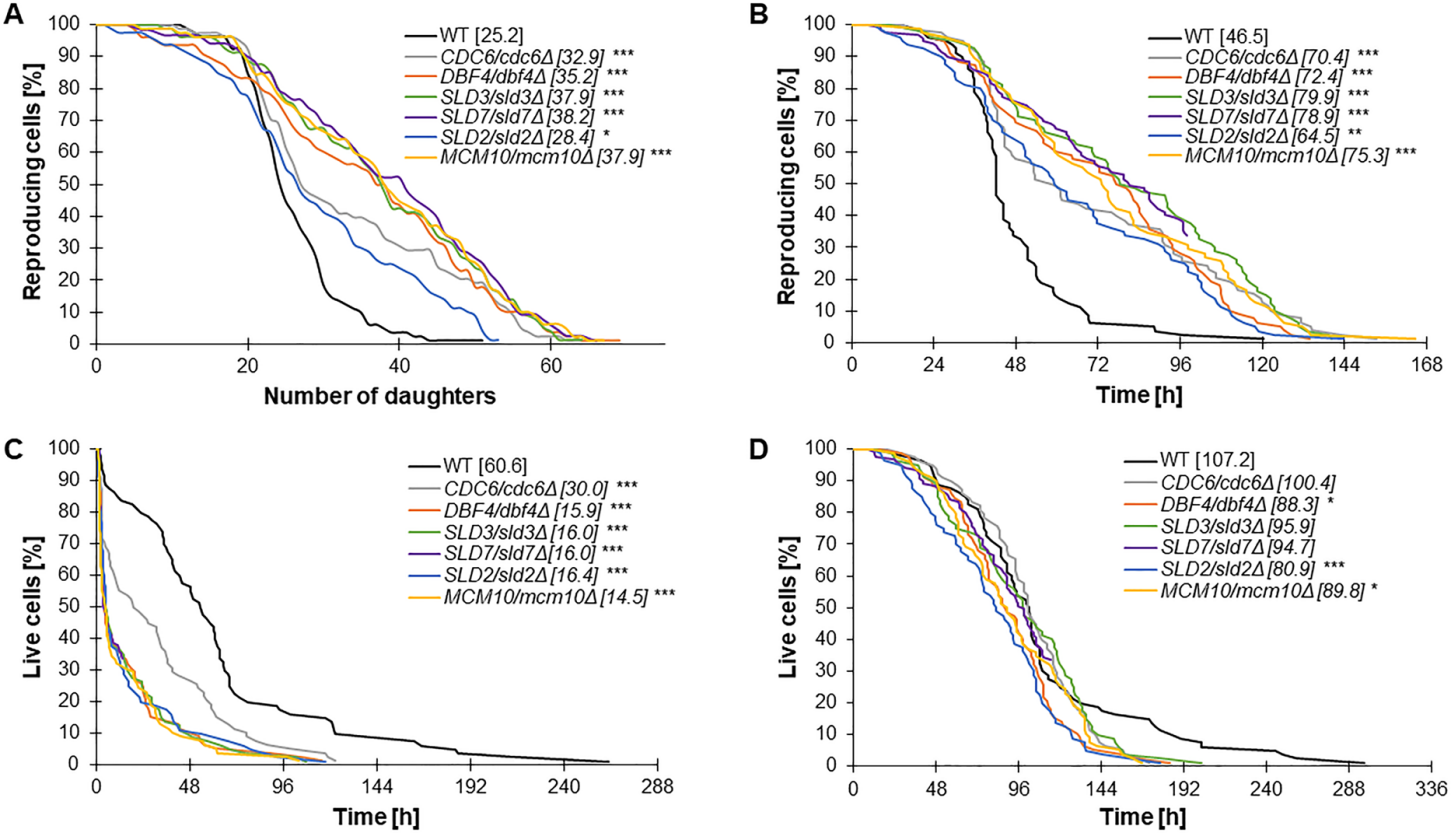
Aging phenotypes of the lowPICC strains. Comparison of the reproductive potential (**A**), reproductive lifespan (**B**), post-reproductive lifespan (**C**) and total lifespan (**D**) of the diploid BY4743 (WT) and isogenic heterozygous strains. Statistical significances were assessed using ANOVA and Dunnett’s post hoc test (*p < 0.05, **p < 0.01, ***p < 0.001). The mean value for a total of 90 cells from two independent experiments is shown in parentheses

Our data clearly showed that heterozygous loci presence in the analyzed strains impacts the aging of both active mitotically and post-mitotic cells. As shown in Fig. 5A, all strains had significantly extended reproductive potential (*p* < 0.001). In almost all heterozygous strains (except *SLD2/sld2Δ*), the mean budding lifespan exceeded 30 doublings performed by a single yeast mother cell. The highest average reproductive potential had the *SLD7/sld7Δ* (38.15), *MCM10/mcm10Δ* (37.9), *SLD3/sld3Δ* (37.9) and *DBF4/dbf4Δ* (mean 35.2 doublings/cell) strains. In particular, it is worthwhile to highlight the maximum number of doublings performed by individual cells. The WT is the only strain that has performed a maximum of 50 doublings, while almost all of the analyzed heterozygotes have performed a maximum between 65 and 70 doublings (exception *CDC6/sld2Δ* and *SLD2/sld2Δ*) (Fig. 5A).

Based on Minois’ concept, yeast cells do not die after the last doubling (Minois et al. 2005). This key observation allows the introduction of the time parameter in yeast aging analyses. The total lifespan was introduced and consists of two phases, reproductive and post-reproductive, which may be regulated differently. A significant increase in the reproductive potential (Fig. 5A) with an additional significant increase in the doubling time (Fig. 1D) leads to an increase in the reproductive time (reproductive lifespan). Figure 5B shows a significant increase in the reproductive lifespan in all analyzed strains compared to the WT. Post-reproductive lifespan refers to the last phase of a yeast cell’s life, i.e., from endings of budding to cell death. In Fig. 5C, we showed a significant decrease in the post-reproductive lifespan of all analyzed heterozygotes compared to WT (*p* < 0.001). The extremely short mean post-reproductive lifespan was observed in the case of *DBF4/dbf4Δ*, *SLD2/sld2Δ*, *SLD3/sld3Δ*, *SLD7/sld7Δ* and *MCM10/mcm10Δ* heterozygous strains: it was approximately four times shorter in comparison to WT. In turn, total lifespan is determined as the sum of time (hours) that cells spend in the reproductive and post-reproductive phases of life. Interestingly, as shown in Fig. 5D, only one copy of the respective gene in lowPICC strains led to a decrease in the total lifespan of all analyzed strains compared to WT. A statistically significant acceleration of aging was observed for the *SLD2/sld2Δ* (*p* < *0.001*), *DBF4/dbf4Δ* (*p* < 0.05) and *MCM10/mcm10Δ* (*p* < 0.05). As shown in Fig. 5D, a significant difference was observed also in the maximal survival time of heterozygous cells compared to WT. We found that all tested strains had a shorter maximum lifespan (about 100 h) than WT. The previous analyses performed using strains heterozygous with respect to genes encoding proteins involved in replication initiation showed no effect on total lifespan, making the obtained results surprising (Stepien et al. 2022). Here, we demonstrated that reduced expression of *DBF4*, *CDC6*, *SLD2*, *SLD3*, *SLD7* and *MCM10* genes also affects the total lifespan of mitotically active cells, which is a novelty in yeast aging research.

**Fig. 6:**
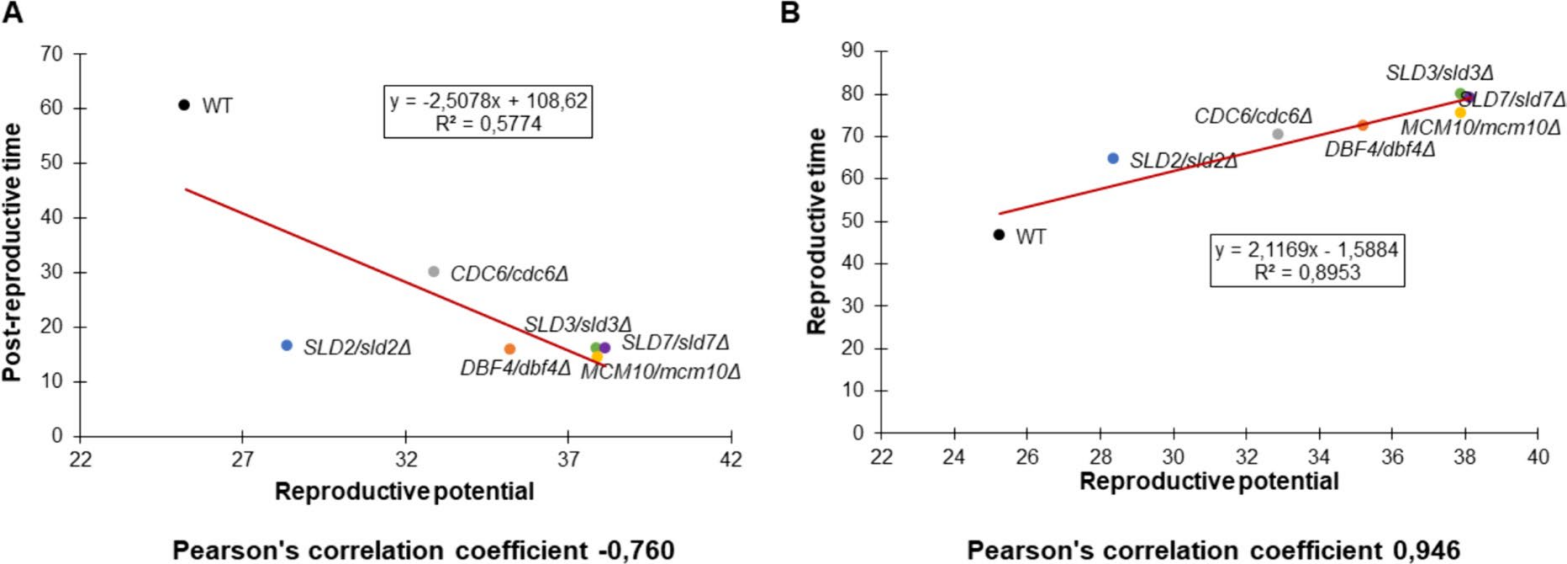
Comparison between mean reproductive potential and mean reproductive lifespan according to Pearson’s correlation coefficient (**A**) and between the mean reproductive potential and mean post-reproductive lifespan (**B**) of the WT strain (BY4743) and the isogenic heterozygous lowPICC strains

Then, we showed the correlation between the selected aging parameters (Fig. 6). As visible in Fig. 6A, a negative correlation between post-reproductive lifespan and the reproductive potential is evident. The trend line suggests a strong negative correlation between these parameters, and the value of the Pearson correlation coefficient is − 0.76. Here, we also presented a strong positive correlation between the reproductive lifespan and the reproductive potential (Pearson correlation coefficient is 0.946) (Fig. 6B). This suggests that there is a trade-off between the reproductive lifespan and post-reproductive lifespan. In other words, as the reproductive lifespan increases, the post-reproductive lifespan decreases. This is likely due to the fact that organisms are allocating more resources to reproduction and less to maintenance and repair. Even though yeast is a single-celled organism, the mechanisms of replicative aging share certain similarities with the aging processes in multicellular organisms, including humans. Therefore, understanding this process in yeast can provide insights into the general mechanisms of cellular aging and potential strategies to delay this process.

A different method to measure yeast lifespan has been called CLS (Fabrizio and Longo 2003; Longo et al. 1996). Therefore, this method assesses how long yeast cells can survive and remain active in a non-budding phase. Living cells (survival) are counted by measuring their ability to clonogenicity (CFU) during growth on a rich YPD solid medium. In synthetic dextrose media, cell density achieves its maximum after 2–3 days, but the metabolic state remains high for up to 6 days after that (Gray et al. 2004). With these methods, it has been demonstrated that yeast cells can survive up to a period of weeks in a non-budding state, which can provide a valuable source of knowledge in studies aimed at understanding aging biology. A CLS measures the survival of non-budding cells and can also be used as a model for aging in post-mitotic high eukaryote cells, including humans. This makes the CLS an ideal tool for studying aging in non-dividing cells such as human cells.

Overall, the CLS was found to be a useful tool for understanding the biological processes of aging and aging-related diseases. All lowPICC strains displayed slowed aging during the first 14 days of the experiment, as shown in Fig. S2. Statistically significant changes were observed on the 4th and seventh day of the experiment (*p* < 0.001). All analyzed strains showed higher survival than WT. It was observed that on the 14th day, *SLD3/sld3Δ*and *SLD7/sld7Δ* exhibited the highest survival rates, with values of ~ 35% and ~ 25%, respectively. In turn, the lowest survival rate in the analyzed group was characterized by the *CDC6/cdc6Δ* strain (15%). These findings suggest that *SLD3/sld3Δ* and *SLD7/sld7Δ* may be potential candidates for further investigation.

Our previous report suggests that cells’ size increased successively during chronological aging (Stepien et al. 2022, 2020). Consequently, the smallest cells were observed at the starting point of the experiment (2nd day), and the largest ones were observed on the final step of the experiment (14th and 21st day). Here, we confirm a ploidy reduction with time in all analyzed strains and the WT control (Fig. S3). Our results reveal that autophagy is a key factor determining the ploidy reduction of chronological aging cells (Enkhbaatar et al. 2023).

### Lowered level of lowPICC causes a change in the biochemical fingerprint

**Fig. 7:**
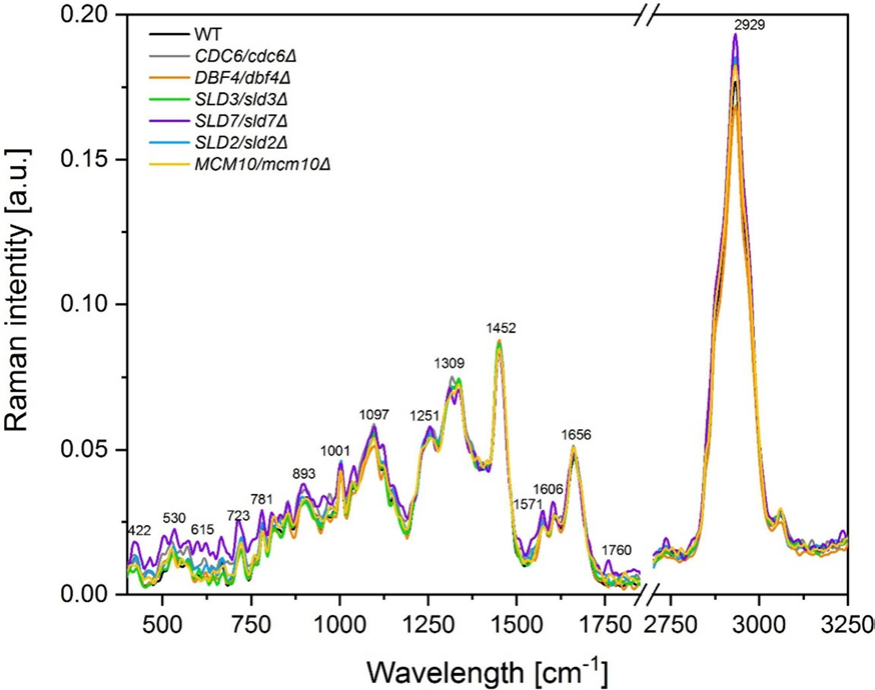
Raman spectra of the yeast (WT and lowPICC strains) with the regions corresponding to vibrations of functional groups

The Raman spectra for all analyzed yeast lowPICC strains are presented in Fig. 7. In these spectra, the peaks are found at the same positions (Raman shift) but with different intensities for all the analyzed samples.

The peaks correspond to vibrations of functional groups in the proteins, lipids, polysaccharides, and RNA (Table 3). The differences in peaks’ intensities are explained by various amounts of chemical compounds, which show changes in the metabolic composition of the presented yeast mutants. In the presented Raman spectra, the most differences were visible in bands characteristic of lipids (1309, 1760, and 2929 cm^−1^), polysaccharides (422, 522, 893, and 970 cm^−1^), RNA (530–723, and 1571 cm^−1^), and proteins (1039 and 1606 cm^−1^). The Raman spectrum of the *SLD7/sld7Δ* strain is characterized by bands of the highest intensity and additional bands (522, 530, and 1760 cm^−1^), which were not observed in other lowPICC strains and WT strains.

**Table 3:** Listing of the positions of the Raman bands identified in yeast sample with the description of vibrations corresponding to the respective functional groups

Vibrations	Functional groups	Range of peak positions [Raman shift, cm^−1^]
CH3/CH2 twisting, wagging and/or bending, C = C, C = O, CH_2_ asymmetric stretch	lipids	1251, 1309, 1656, 1760, 2929
ν_2_ PO_4_^3−^, C–C skeletal	polysaccharides	422, 522, 893, 970, 1097
ν(C–C) of proteins	proteins	854, 1001, 1039, 1452, 1606
C–C bending mode of phenylalanine, ν(O–P–O) RNA, C-OH_3_, ring breathing,	RNA	499, 530, 565, 600, 615, 665, 723, 781, 1332, 1571

In turn, the PCA analysis showed differences between analyzed strains within the functional groups studied (lipids, polysaccharides, proteins, and RNA). Two yeast heterozygous strains (*SLD7/sld7Δ* and *CDC6/cdc6Δ*) significantly differed in their chemical composition from the other strains.

**Fig. 8:**
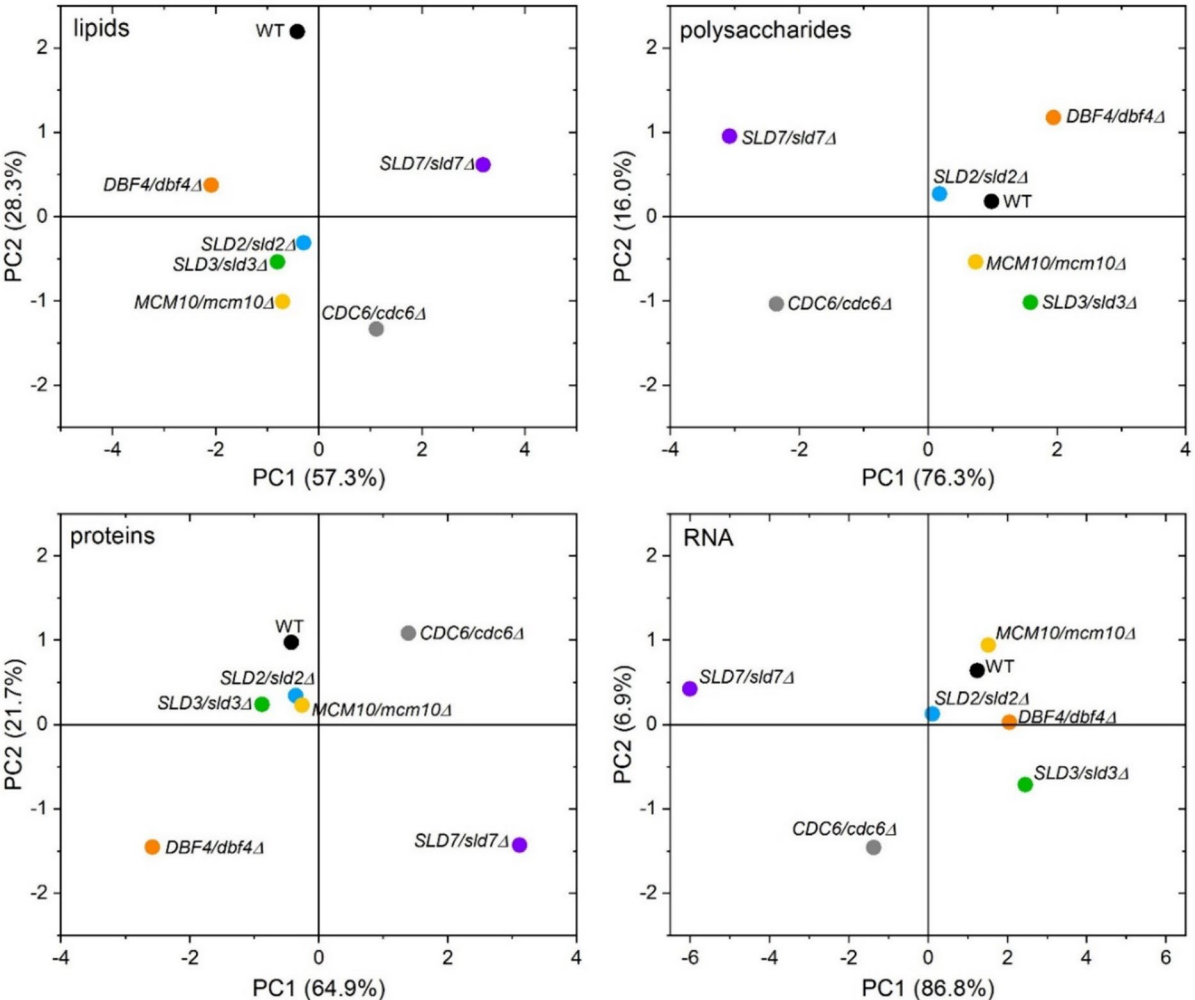
The 2D score graphs for PCA of FT-Raman data show the relationships between analyzed yeast (WT and lowPICC strains) within the identified functional groups (lipids, polysaccharides, proteins, and RNA)

The PCA analysis distinguished them as separate points in all tested functional groups. In the case of proteins, three strains (*SLD7/sld7Δ*, *CDC6/cdc6Δ*, and *DBF4/dbf4Δ*) stood out from the rest of the analyzed strains (Fig. 8). In conclusion, interestingly, they lack one copy of the *SLD7* gene significantly affected the metabolism of *SLD7/sld7Δ* cells, distinguishing it from other lowPICC strains and WT control (Fig. 7). This was indicated by both the Raman spectrum and the PCA analysis.

**Fig. 9:**
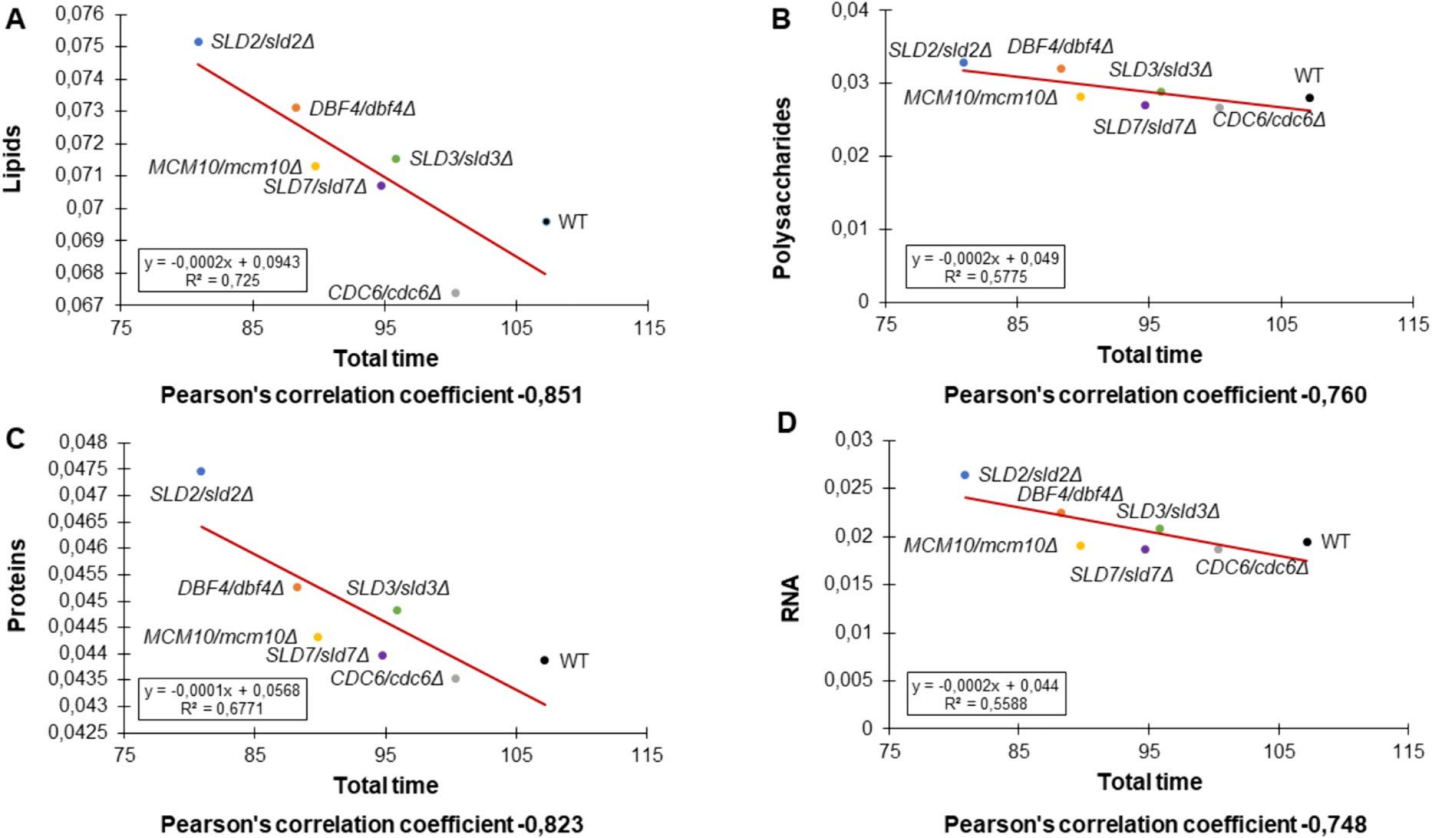
Comparison between mean total lifespan and lipids (**A**), polysaccharides (**B**), proteins (**C**), and RNA (**D**) contents according to Pearson’s correlation of the WT strain (BY4743) and the isogenic heterozygous lowPICC strains

The correlations between the content of macromolecules in cells and aging appear interesting. We discovered a strong negative correlation between the content of lipids, proteins, RNA, and polysaccharides and the lifespan of cells (Fig. 9A–D). This means that maintaining the content of these macromolecules in the cell at a relatively low level ensures the maintenance of the aging rate at the level of the WT strain. On the other hand, the accumulation of these macromolecules in the cell leads to accelerated aging and cell death, which was observed in *SLD2/sld2Δ* and *DBF4/dbf4Δ* strains.

These results indicate the key importance of the content of proteins, lipids, polysaccharides, and RNA on aging in mitotically active yeast cells. These results also underline that maintaining the proper balance of macromolecules in the cell is necessary to maintain longevity.

Lipids perform a wide range of biological functions, from keeping membranes structurally intact to providing energy storage and signaling. Lipids also play a significant role in the aging process of yeast cells (Beach et al. 2013). The connection of lipids to cell death is complex, and so far, it is been poorly understood. Studies in yeast have revealed various aspects of lipotoxicity, including the toxicity of free fatty acids, cell death modulated by sphingolipids, as well as the involvement of lipid peroxidation in the mitochondrial pathways of apoptosis (Eisenberg and Büttner 2014). Some recent studies have demonstrated that the regulation of specific lipid species plays an important role in the process of human aging. It has been reported in several studies that senescent cells accumulate more lipid droplets than proliferating cells. Therefore, in senescent cells, deregulated lipid accumulation may be a result of increased lipid uptake, an increase in lipid biosynthesis pathways, or a deregulation of lipid breakdown pathways (Chee et al. 2021; Flor et al. 2017).

**Fig. 10:**
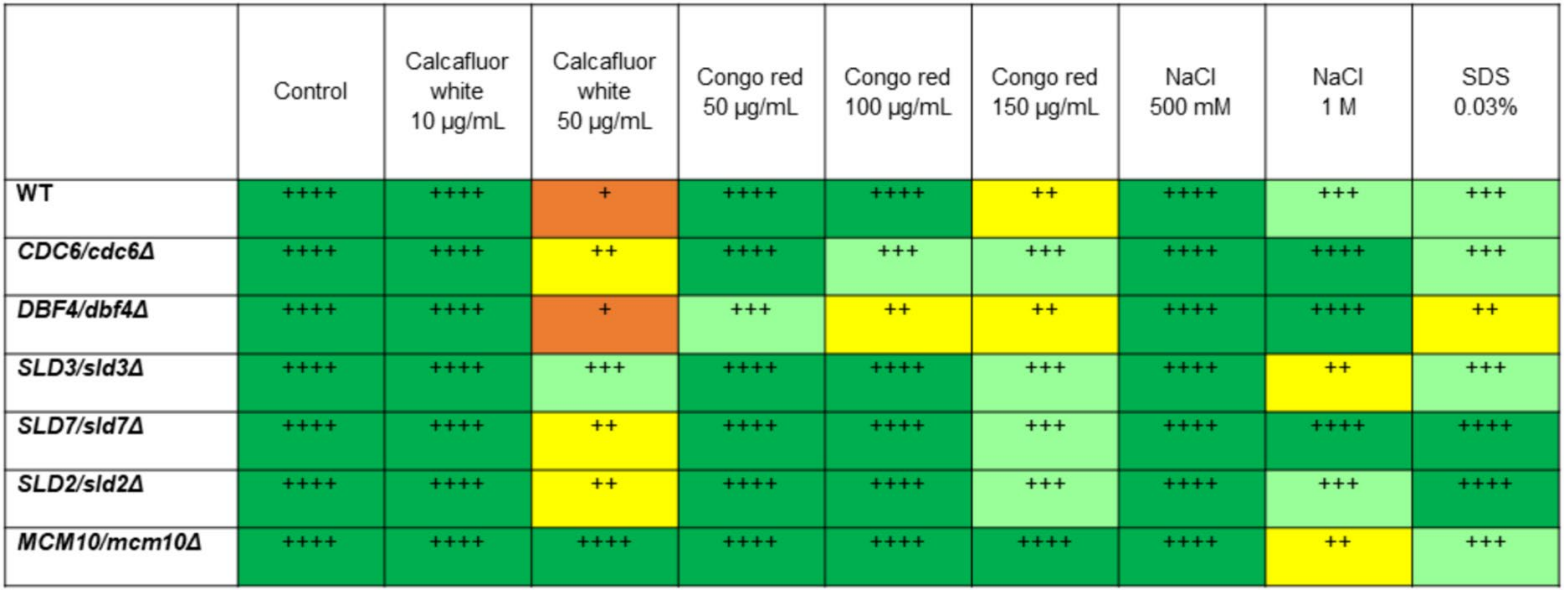
Drug sensitivity analysis results were performed for lowPICC strains, using drop test assay

Polysaccharides in yeast are found mainly in the cell wall (Saadat et al. 2021). The role of polysaccharide content in yeast aging has not been analyzed so far. However, we have previously reported that the cell wall is a key factor determining the reproductive potential of the cell and probably longevity (Molon et al. 2020, 2018). Interestingly, the analyzed strains showed increased resistance to cell wall inhibitors (Congo red and Calcofluor White) compared to WT (Fig. 10). This indicates that the proteins involved in the initiation of DNA replication also play a role in cell wall biogenesis and the adaptation of the yeast cells to changing environmental conditions, including stress.

The link between cellular protein levels and yeast aging is a topic of broad research. In yeast, prior studies have demonstrated that disruptions in ribosome structure, which slow down translation, contribute to the deceleration of aging (Borkiewicz et al. 2019; Steffen et al. 2008, 2012). Conversely, an increase in the quantity of ribosomal proteins accelerates aging and diminishes the cell’s reproductive potential (Molon et al. 2023). Therefore, proteostasis, or protein homeostasis, refers to the healthy maintenance of the cellular proteome and involves highly complex and interconnected pathways that govern the fate of proteins from synthesis to degradation. It is well-recognized that the ability of cells to maintain proteostasis declines during aging (Sampaio-Marques and Ludovico 2018). We have a hypothesis that the accumulation of proteins in cells of accelerated aging strains may be related to the deregulation of two main degradation pathways, i.e., ubiquitin–proteasome system and autophagy.

The stability and metabolism of RNA, including its levels, have been linked to cell life and aging in yeast. However, it is important to note that the relationship between RNA levels and longevity in yeast is complex and multifaceted (Falcone and Mazzoni 2018). Exact mechanisms and relationships between RNA levels and longevity in yeast are still an active area of research. Interestingly, the analyzed strains exhibited a significantly prolonged doubling time, yet their lifespan was shorter compared to the WT. We have previously demonstrated that a decrease in metabolic rate, coupled with an increase in doubling time, is strongly correlated with longevity (Molon et al. 2016). Elevated RNA levels, in general, may suggest various factors, including potential impairment of ribosome assembly.

Among the analyzed strains, *SLD2/sld2Δ* is particularly noteworthy. Despite its reproductive potential being slightly higher than that of the WT, it exhibits the most rapid aging. The improper response to DNA damage (suggested by the increased number of Rad52 foci during genotoxic stress and accumulation of mtDNA in these cells) is visible even though among all tested lowPICC strains, the expression of a respective gene from heterozygous locus reached the highest level (63% of control strain level versus e.g., 15% observed for *MCM10* gene). Thus, the maintenance of cellular homeostasis is critical for yeast longevity, and disruptions in this balance can accelerate the aging process.

There are many unknowns in the biology of aging. In a recently published article by Suresh Rattan, the author tried to tide up the knowledge concerning aging and indicate the gaps in the biogerontology field (Rattan 2024). We believe the yeast research may help answer some questions posed in that article, e.g., about the evolved public (universal) and private (species-specific) longevity assurance genes for the essential lifespan of a species. Our data demonstrated the necessity of the trade-off between reproductive lifespan and post-reproductive lifespan. Organisms seem to allocate more resources to reproduction and less to maintenance and repair. This strategy might explain the reset clock of reproductive potential in young cells and mitotic catastrophe in proliferatively old cells.

In summary, our data unequivocally show that a reduction in the copy number of genes encoding proteins involved in the regulation and/or initiation of DNA replication influences the acceleration of aging in mitotically active yeast cells and delays the aging of post-mitotic cells. All of the strains analyzed show a significantly extended reproductive potential, which may be associated with a subtle disruption of the cell cycle and an extension of the doubling time. Importantly, here we demonstrate a strong negative correlation between the content of cellular macromolecules (RNA, proteins, lipids, polysaccharides) and aging. The data we obtained show that disturbances in the initiation of genomic DNA impact not only the cell cycle or the doubling time of the cell but also the entire biochemical profile of the cells.

## Supplementary Information

Below is the link to the electronic supplementary material.

Supplementary file1 (DOCX 877 KB)

## Data Availability

No datasets were generated or analysed during the current study.
