# Supplementary material for: Restricting the level of the proteins essential for the regulation of the initiation step of replication extends the chronological lifespan and reproductive potential in budding yeast
Source: Biogerontology. 2024 Jun 6;25(5):859–81. doi: 10.1007/s10522-024-10113-x (PMC11374879; doi:10.1007/s10522-024-10113-x)
Supplement: Supplementary file 1 — Supplementary file1 (DOCX 877 KB) [file 10522_2024_10113_MOESM1_ESM.docx]

Supplementary data





Figure S1. DNA content analysis of lowPICC strains. Flow cytometry analysis of the haploid (BY4741) and diploid (BY4743) WT strains used as controls (A) and the isogenic heterozygous strains *DBF4/dbf4Δ*, *CDC6/cdc6Δ*, *SLD7/sld7Δ*, *SLD2/sld2Δ*’ *SLD3/sld3Δ*, *MCM10/mcm10Δ* (B) was performed. The representative histograms from three biological repetitions are presented. On the histograms, the gating conditions used to calculate the number of cells in the individual cell cycle phases were shown.


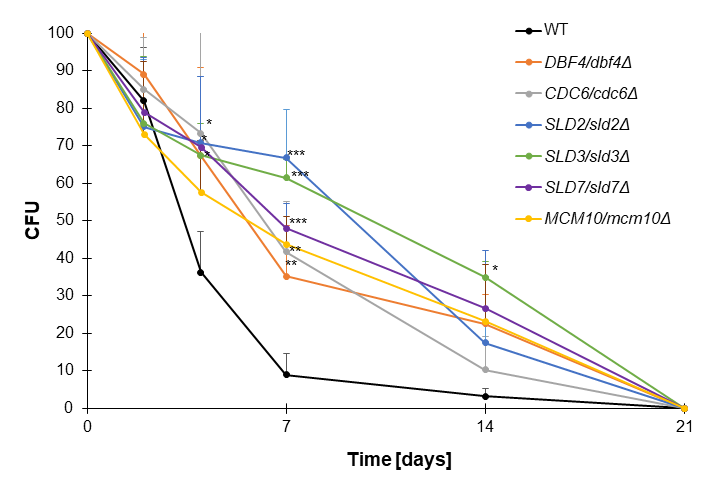


Figure S2. Chronological lifespan of the diploid WT (BY4743) and the isogenic heterozygous strains *CDC6/cdc6Δ*, *DBF4/dbf4Δ*, *SLD2/sld2Δ*, *SLD3/sld3Δ*, *SLD7/sld7Δ* and *MCM10/mcm10Δ.* Survival was determined by a colony-forming units (CFU) assay. Error bars represent standard deviations obtained from three independent experiments. Statistical significance was assessed using ANOVA and Dunnett’s post hoc test (* p < 0.05, ** p < 0.01, *** p < 0.001).


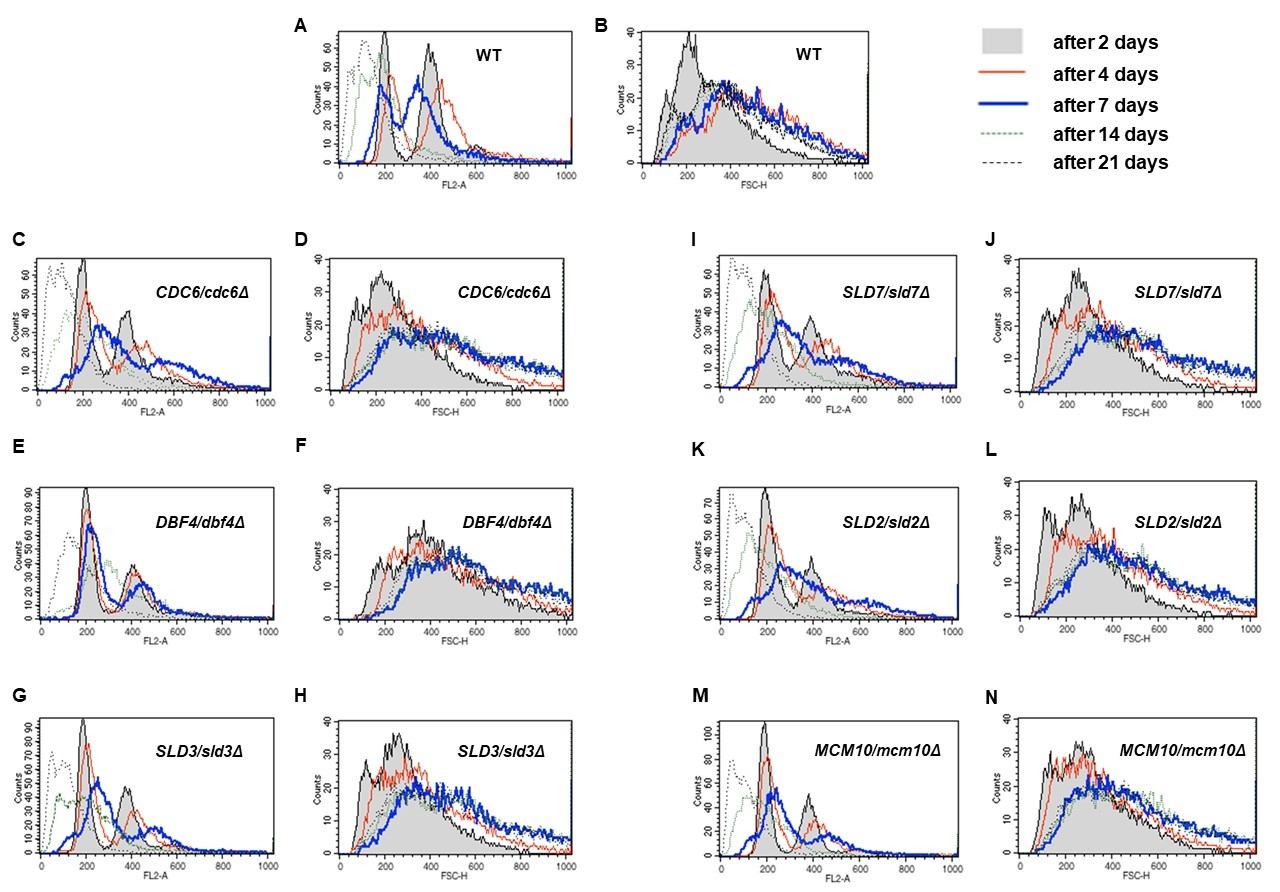


Figure S3. Ploidy reduction and cell size increase accompany chronological aging. Results of the DNA content analysis via flow cytometry during the chronological lifespan analysis at selected time points (A, C, E, G, I, K, M). For the DNA content analysis, yeast cells were stained with propidium iodide. The representative histograms are shown. Three independent experiments were performed. Cell size of the WT diploid (BY4743) strain and the isogenic *DBF4/dbf4Δ*, *CDC6/cdc6Δ*, *SLD7/sld7Δ*, *SLD2/sld2Δ*’ *SLD3/sld3Δ*, *MCM10/mcm10Δ* strains during the chronological lifespan analysis in selected time points (B, D, F, H, J, L, N). Cell size as measured by forward scatter (FSC histogram reflects the cells size in the assayed population). The cells (10,000 cells per sample) were analyzed via flow cytometry as described in the Materials and Methods section.
